# 3D fractional moving blood volume (3D-FMBV) demonstrates decreased first trimester placental vascularity in pre-eclampsia but not the term, small for gestation age baby

**DOI:** 10.1371/journal.pone.0178675

**Published:** 2017-06-01

**Authors:** Sally L. Collins, Alec W. Welsh, Lawrence Impey, J. Alison Noble, Gordon N. Stevenson

**Affiliations:** 1 The Nuffield Department of Obstetrics and Gynaecology, University of Oxford, Oxford, United Kingdom; 2 The Fetal Medicine Unit, John Radcliffe Hospital, Oxford, United Kingdom; 3 School of Women’s and Children’s Health, University of New South Wales, Randwick, New South Wales, Australia; 4 Department of Maternal-Fetal Medicine, Royal Hospital for Women, Randwick, New South Wales, Australia; 5 The Institute of Biomedical Engineering, Department of Engineering Science, University of Oxford, Oxford, United Kingdom; Stellenbosch University, SOUTH AFRICA

## Abstract

**Objective:**

To undertake an observational study to see whether first trimester placental vascularity, measured with a standardized power Doppler index: 3D-FMBV, is different in pregnancies which either develop pre-eclampsia or lead to term, normotensive small for gestational age (SGA) babies.

**Methods:**

Women were scanned between 11 and 13^+6^ weeks. The placental volume (sPlaV) was estimated using our previously validated semi-automated tool. Estimates of 3D-FMBV were generated from the raw power Doppler signal for the whole utero-placental interface, UPI (FMBV-UPI) and 5mm into the placenta (FMBV-IVS). Differences in the placental volume and FMBV for pregnancies developing pre-eclampsia and resulting in term, normotensive SGA babies were compared with term, normotensive, appropriate for gestational age (AGA), controls.

**Results:**

Results were available for 143 women. The placental volume (sPlaV) was reduced in both pre-eclampsia (p = 0.007) and term, normotensive SGA (p = 0.001) when compared with term normotensive AGA controls. 3D-FMBV estimates were significantly lower for pregnancies developing pre-eclampsia (FMBV-UPI, p = 0.03, FMBV-IVS, p = 0.01) but not for the normotensive SGA pregnancies (FMBV-UPI, p = 0.16, FMBV-IVS, p = 0.27).

**Conclusion:**

Pregnancies destined to develop pre-eclampsia are more likely to have small placentas with significantly reduced vascularity at 11–13 weeks. Those pregnancies which were normotensive throughout but resulted in an SGA baby delivered at term, had significantly smaller placentas but with similar vascularity to normotensive AGA pregnancies.

## Introduction

Pre-eclampsia and small for gestation age (SGA) babies, defined as <10th centile on customised growth charts[1] [2, 3], contribute disproportionately to the number of stillbirths worldwide [4, 5]. These babies are also at increased risk of other adverse perinatal outcomes [6–9]. Although much work is underway to find a first trimester screening test for pre-eclampsia [10, 11] little appears to be available to screen for SGA. Developing a simple, robust early screening test to identify those babies at risk of being SGA would help to focus limited healthcare resources towards increased surveillance of these pregnancies and potentially decrease perinatal mortality and morbidity rates.

Differences in the haemodynamics of single spiral arteries at the utero-placental interface (UPI) have been demonstrated with ultrasound (US) in SGA pregnancies [12]. However, the length of time and training required to perform this measurement means it is not feasible to be used for screening. Instead, a more robust and rapid assessment of vascularity that simultaneously images and analyses the spiral arteries feeding the whole placental bed (i.e. capturing the whole UPI and inter-villous space (IVS) in 3D) would be a highly desirable tool with potential for clinical utility. With its ability to estimate the degree of vascularity in tissue, 3D power Doppler (PD) ultrasound appears to be the ideal image modality to provide this. Until now, the only available quantitative measures of ‘vascularity’ have been commercially generated indices provided by the machine manufacturers. The validity and exact meaning of these indices have been hotly debated [13–15] dividing opinion as to their usefulness. What is clear though, is that to make meaningful, quantitative inter-patient comparison, the differences in tissue attenuation unique to each clinical situation needs to be controlled for [16]. The PD measurement, fractional moving blood volume (FMBV) is the only tool that is able to do this and has been validated in 2D in animal studies [17, 18]. This technique cannot be performed in 3D with any of the commercially available software [13]. Using the raw data exported directly from the machine, we have developed a software tool to enable FMBV to be calculated in three dimensions (3D-FMBV) [19]. This allows standardised measurement of the vascularity of the entire UPI in the first trimester placenta.

This study is the first clinical application of 3D-FMBV, employing it to investigate whether the vascularity at the UPI and in the IVS is altered at 11 to 13^+6^ weeks in pregnancies destined to develop pre-eclampsia and/or be SGA at term.

## Materials and methods

The study was conducted with NHS REC ethical approval (REC ref: 08/H0604/163). Written consent was obtained before enrolment. Women with singleton pregnancies undergoing a first trimester scan at the John Radcliffe Hospital in Oxford, UK over a 12 month period were invited to participate. One third of the participants invited to participate were deemed to be at increased risk of pre-eclampsia and SGA as they had a history of pre-eclampsia or SGA babies in a previous pregnancy. The remaining two thirds were taken from an unselected population. Those under the age of 16 years, those with a BMI >35, or significant maternal chronic illness including diabetes, or treatment with medications associated with fetal growth restriction, such as beta-blockers [20] were not invited to participate in the study. Gestational age was calculated from the CRL at the first visit [21]. Sociodemographic and obstetric data were collected including age, parity, family history, past medical and obstetric histories. The results of the combined screening test were available to the investigators. No data was available to estimate the effect size of the differences in vascularity therefore the sample size was calculated to demonstrate a difference in first trimester placental volume between SGA and AGA pregnancies.

The scans were undertaken by a single operator (SC) with the participant in a semi-recumbent position at a gestation of 11 to 13^+6^ weeks. 3D volumetric scans of the placenta were acquired using a GE Voluson E8™ (GE Healthcare, Milwaukee, WI, USA) and RAB4-8-D 3D/4D curved array abdominal transducer (4–8.5 MHz). After confirmation of viability and identification of placental position, the optimal probe placement for 3D acquisition of the whole placenta was identified. This was usually a cross-sectional plane close to the centre of the placenta. A static PD volume was captured using pre-determined machine settings [22, 23] and an individualised sub-noise gain (SNG) [22]. The volume was then checked to ensure that the whole placenta had been included; if not, the angle was increased or the probe repositioned and the process repeated. If any PD flash artifact was seen the process was repeated. Once a complete 3D PD placental volume had been captured it was stored and analysed off-line. All analysis was performed blinded to the pregnancy outcome.

**Fig 1:**
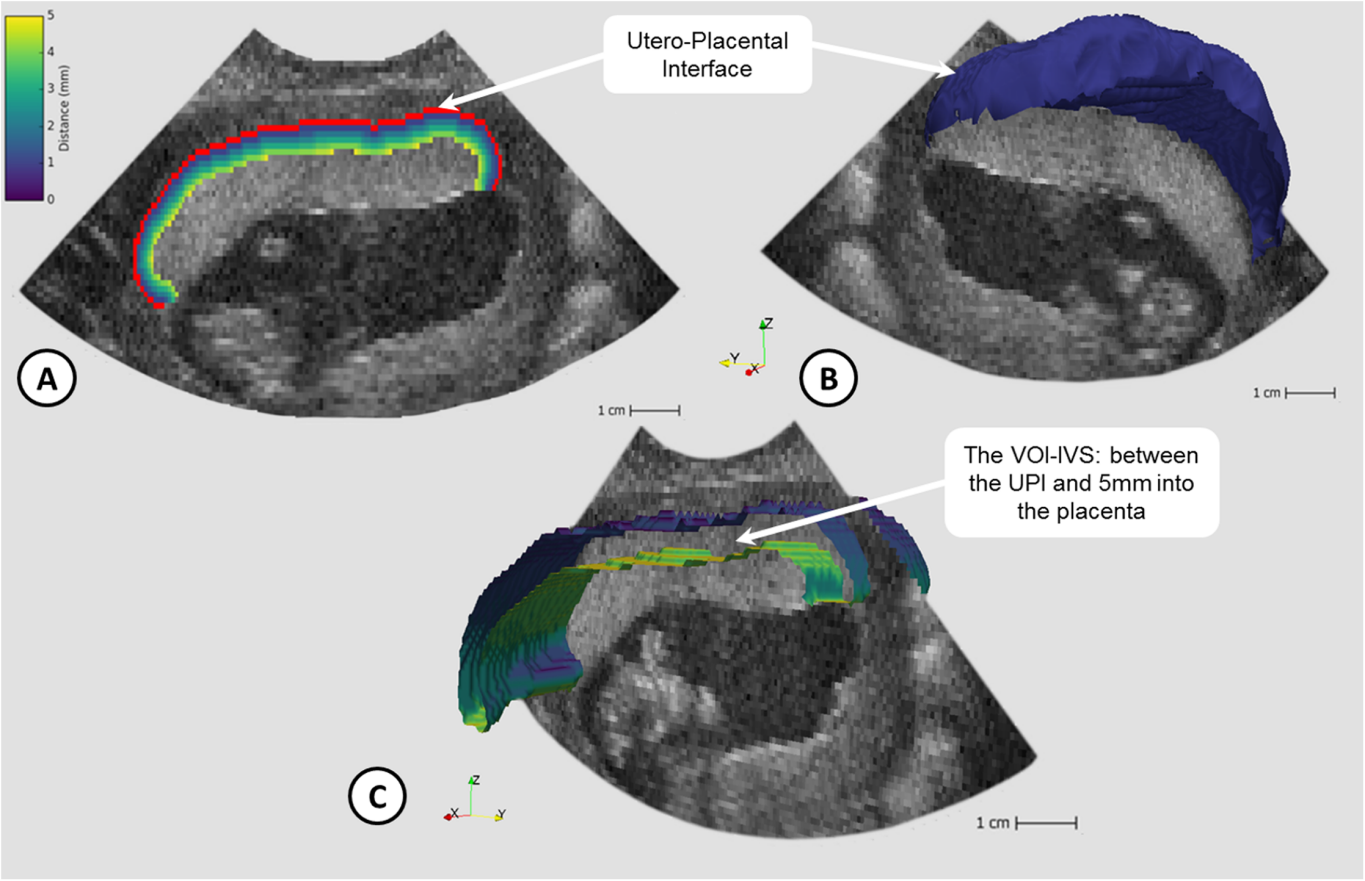
Visualisation of utero-placenta interface (UPI) and volume of interest (VOI-IVS: 3D ‘slice’ of tissue between the UPI and 5mm into the placenta containing the intervillous space (IVS)). Fig 1a) 2D B-Mode image with UPI overlaid (red) and voxels up to 5mm into placenta labelled based on distance; Fig 1b) 3D UPI (blue) shown with 2D B-mode slice; Fig 1c) Section of the 3D VOI-IVS coloured by distance away from UPI.

The 3D placental volume was estimated using our semi-automated image analysis tool [24] and corrected for gestation by comparison with the crown-rump length (CRL) to produce the dimensionless index, standardized placental volume (sPlaV; calculated as (PlaV)^1/3^ divided by CRL) [25]. The estimates of 3D-FMBV were calculated according to the previously reported technique [19] using the raw 3D-PD ultrasound data for the following volumes of interest; the whole utero-placental interface (1mm deep—UPI), and 5mm into the placenta from the UPI (volume containing the intervillous space—IVS), see Fig 1.

Postpartum data were collected by reviewing the hospital notes. The customised birth weight centile was calculated using the Grow™ software package (version 7.5.1, West Midlands Perinatal Institute, Birmingham, UK). Small for gestational age (SGA) was defined as <10th centile on customised birth weight charts (SGA) with appropriate for gestation age (AGA) babies being those ≥10th centile. Pregnancy associated hypertensive disorders including pre-eclampsia, were defined according to the International Society for the Study of Hypertension in Pregnancy (ISSHP) guidelines [26]. Early onset pre-eclampsia was defined as diagnosis of pre-eclampsia before 34 weeks. The Mann-Whitney test was used to detect a difference in the standardised placental volume (sPlaV), FMBV at the UPI (FMBV-UPI) and the FMBV for the volume 5mm into the placenta from the UPI (FMBV-IVS) for; SGA compared to AGA babies in the whole population; SGA compared to AGA in the term, normotensive population and pre-eclamptic pregnancies compared to non pre-eclamptic pregnancies in the whole population. Linear regression analysis was used to test if there was any correlation between FMBV and gestational age.

Statistical analyses were performed using SPSS (version 22, IBM Corporation, NY, USA). Results were considered to be statistically significant when p < 0.05.

## Results

**Fig 2:**
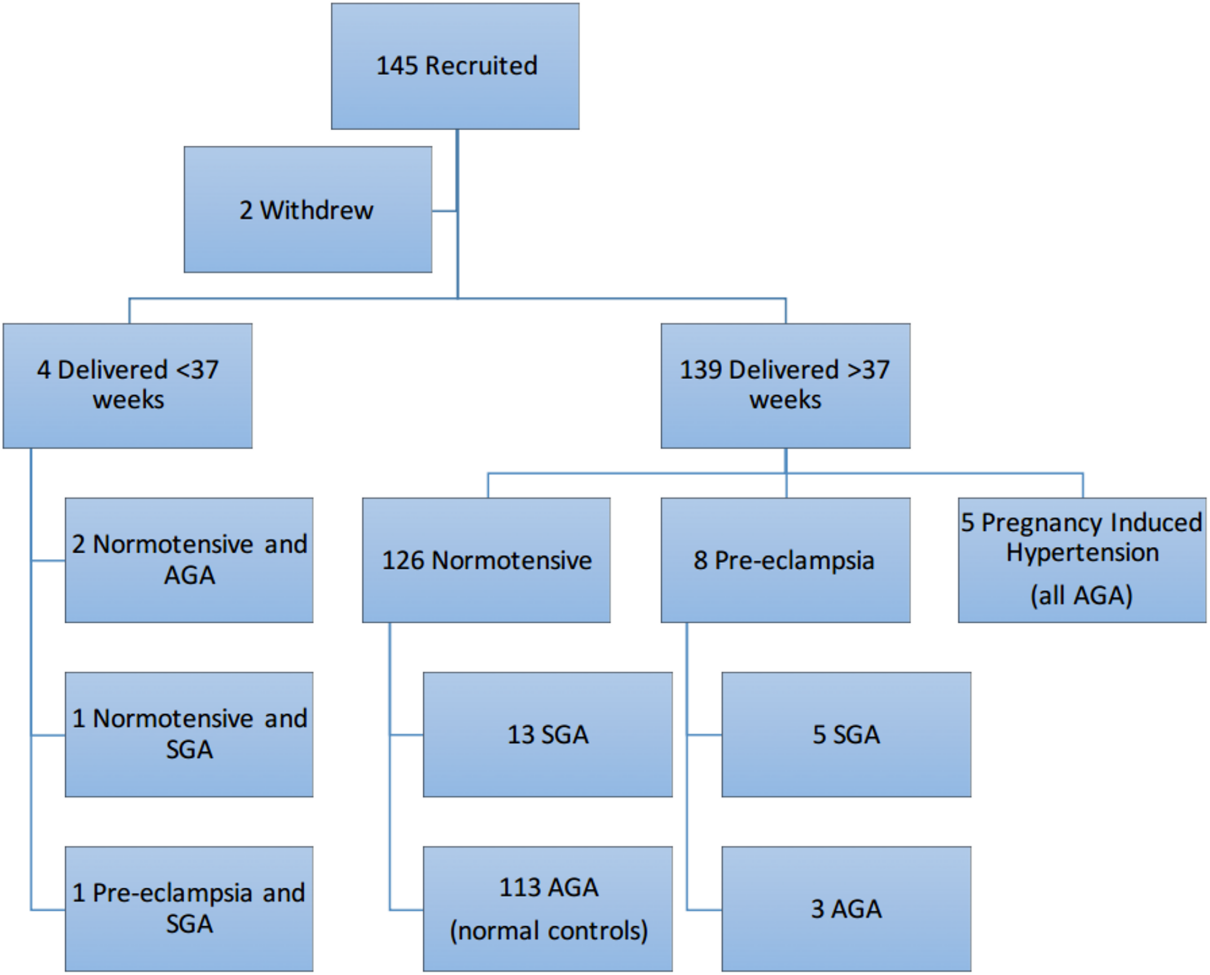
Flow diagram demonstrating the outcomes of all the participants recruited.

One hundred and forty-five wome with a singleton pregnancy were recruited. One participant emigrated and one terminated the pregnancy at 13 weeks after a diagnosis of Trisomy 21, leaving data from 143 women to be analysed. All 143 babies were phenotypically and chromosomally normal. One hundred and thirty-nine women delivered after 37 weeks’ gestation; 126 were normotensive throughout pregnancy; eight developed late onset pre-eclampsia and five were diagnosed with pregnancy induced hypertension. Of the four who delivered before 37 weeks, two were appropriately grown for gestation, one was SGA and one had early onset pre-eclampsia and was SGA. In total there were 20 babies who were SGA on customised centiles. For a full breakdown of the outcomes see Fig 2.

There were no significant differences in the baseline demographics between the 20 SGA and the 123 AGA pregnancies. There were no significant differences in the baseline demographics between the nine pre-eclamptic pregnancies and the other 134 women. Table 1 provides a full summary of the baseline demographics for each of the groups. The raw data containing demographics, outcomes and values for 3D FMBV, placenta volume and sPlaV for the study cohort are provided as supplementary material, S1 Dataset.

**Table 1:** Baseline patient demographics.

	SGA (n = 20)	AGA (n = 123)	*P value*	PET (n = 9)	No PET (n = 134)	*P value*
**Age (years)**	31 (16–39)	30 (18–44)	*0*.45*	32 (24–39)	30 (16–44)	0.5*
**BMI (at booking)**	25 (17–35)	24 (18–33)	0.71*	27 (21–35)	24 (17–33)	0.05*
**Smoker**	1	21	*0*.*93*^*†*^	1	21	1.0^*†*^
**Alcohol (mean units/week)**	1.1 (0–4)	1.3 (0–18)	0.49*	0.7 (0–4)	1.3 (0–18)	0.37*
**Multiparous**	12	59	*0*.*68*^*†*^	6	65	0.32^*†*^
**Ethnicity**						
European	18	115	*1*.*0*^*†*^	8	125	0.49^*†*^
Indian	0	2	^*N/A*^	0	2	^*N/A*^
Pakistani	0	1	^*N/A*^	0	1	^*N/A*^
Mixed	1	4	*0*.*54*^*†*^	1	4	0.28^*†*^
East Asian	1	1	*0*.*3*^*†*^	0	2	^*N/A*^
**IVF pregnancy**	0	11	^*N/A*^	0	11	^*N/A*^

P-values calculated using:

* t-test or

^†^ Fisher’s exact test

SGA = small for gestational age; AGA appropriate for gestational age; PET = pre-eclampsia; No PET = no pre-eclampsia; HX = History of; FHx = family history; IUD = intrauterine death; IVF = in-vitro fertilisation

The standardised placental volume was significantly different for all three groups examined (SGA vs AGA, p<0.001; term, normotensive SGA vs term, normotensive AGA, p = 0.04; pre-eclampsia vs no pre-eclampsia, p = 0.007; see Table 2). The FMBV was significantly lower for both volumes of interest (VOI) examined (FMBV-UPI, p = 0.01, FMBV-IVS, p = 0.01) in the SGA babies compared to the appropriately grown ones. However, when the term normotensive SGA babies (n = 13) were compared with the term, normotensive AGA babies (n = 112) the FMBV was not significantly different (FMBV-UPI, p = 0.3, FMBV-IVS, p = 0.4). When the pre-eclamptic pregnancies (n = 9) were compared with the non pre-eclamptic pregnancies (n = 134), the FMBV was significantly different (FMBV-UPI, p = 0.03, FMBV IVS, p = 0.01). FMBV was not correlated with gestational age (FMBV-UPI, R^2^ = 0.005; FMBV IVS, R^2^ = 0.004).

**Table 2:** Comparison by pregnancy outcome.

	All SGA (n = 20)	All AGA (n = 123)	*P value*	Normotensive, term SGA (n = 13)	Normotensive, term AGA (n = 113)	*P value*	PET (n = 9)	No PET (n = 134)	*P value*
Gestation at scan (days)*	93 (83–97)	91 (82–97)	***0*.*4***	94 (83–97)	92 (82–97)	***0*.*2***	89 (83–96)	92 (82–97)	***0*.*5***
CRL (mm)*	70 (53–83)	70 (51–84)	***0*.*4***	76 (57–83)	70 (51–84)	***0*.*2***	64 (53–81)	70 (51–84)	***0*.*4***
**Placental Volume (mm**^**3**^)*	49.1 (14.9–95.0)	60.6 (13.4–130.6)	***0*.*02***	53.1 (26.3–95.0)	61.7 (13.4–130.6)	***0*.*2***	38.7 (14.9–74.1)	60.0 (13.4–130.6)	***<0*.*01***
sPlaV*	0.24 (0.08–0.38)	0.30 (0.08–0.60)	***<0*.*01***	0.26 (0.15–0.38)	0.30 (0.08–0.60)	***0*.*04***	0.20 (0.08–0.30)	0.30 (0.08–0.60)	***<0*.*01***
FMBV UPI*	0.15 (0.03–0.57)	0.24 (0.01–0.65)	***0*.*01***	0.17 (0.06–0.57)	0.24 (0.01–0.65)	***0*.*3***	0.12 (0.03–0.35)	0.23 (0.01–0.65)	***0*.*03***
FMBV IVS*	0.03 (<0.01–0.39)	0.10 (<0.01–0.44)	***0*.*01***	0.09 (0.02–0.39)	0.1 (<0.01–0.44)	***0*.*4***	0.02 (<0.01–0.29)	0.1 (<0.01–0.44)	***0*.*01***

* Median (range)

SGA = small for gestational age; AGA appropriate for gestational age; PET = pre-eclampsia; No PET = no pre-eclampsia; CRL = crown rump length; sPlaV = standardised placental volume ((PlaV)1/3/CRL); FMBV UPI = Fractional moving blood volume at the utero-placental interface (UPI); FMBV IVS = Fractional moving blood volume for the volume containing the intervillous space (IVS), 5mm into the placenta from the UPI.

## Discussion

This is the first time that 3D-FMBV has been calculated from raw US data and applied to a clinical situation. Using this technique, we were able to examine the vascularity of the UPI and the IVS demonstrating that the vascularity in this area is significantly decreased in the first trimester placenta destined to result in a pre-eclamptic pregnancy. The vascularity is not significantly different in the pregnancies destined to produce a term, normotensive SGA baby. However, the first trimester placental volume was significantly smaller for pre-eclamptic pregnancies and for term, normotensive SGA babies. From this sample it would appear that pregnancies destined to develop pre-eclampsia have small placentas with abnormal vascularity at 11–13^+6^ weeks. Those pregnancies which will be normotensive but result in an SGA baby delivered at term, have small placentas but with normal vascularity.

Histopathological studies have demonstrated shallow trophoblast invasion of the spiral arteries with inadequate remodelling in pregnancies complicated by both growth restriction [27–29] and pre-eclampsia [30]. What is not clear however, is if these two pregnancy outcomes actually represent different clinical manifestations of the same underlying pathology [27, 31]. The reduced remodelling and smaller spiral arteries seen histologically should lead to a decreased amount of blood flowing into the intervillous space and therefore should be reflected in the observed vascularity of the IVS. If inadequate spiral artery changes are present in both SGA and pre-eclamptic placentas our findings would suggest that it is only severe enough to significantly reduce the vascularity of the IVS in the first trimester for pregnancies destined to develop pre-eclampsia.

Due to its availability and excellent safety record PD ultrasound is the ideal choice for volumetric imaging the placenta, but the inability to standardise the PD signal using commercially available indices and the flawed method of using a fixed PD gain setting has hampered its development as a clinical tool. These problems are resolved using 3D FMBV and the SNG setting thereby making 3D PD US an affordable non-invasive method for evaluating tissue vascularity. FMBV is able to control for differences in signal attenuation by calibrating the observed power Doppler signal to an area of known maximum signal strength, e.g. a large blood vessel at a similar depth as the target VOI. The influence of certain machine settings and differences in signal attenuation resulting from individual patient characteristics is minimized via this use of this internal control [32]. As FMBV is expressed as standardised percentage vascularity of the volume sampled, it also corrects for any differences in the size of the target. This means that it is appropriate to compare the absolute FMBV even when the placentas are different in absolute size.

We acknowledge limitations of this study. Small for gestational age (SGA) and fetal growth restriction are different, however there is little consensus on diagnosis of growth restriction at term. Customised growth charts were used to adjust for constitutional determinants of birth weight, as they have been shown to represent babies at much greater risk of adverse pregnancy outcomes [2, 33–35]. Our main aim was to identify the ‘at risk’ pregnancy therefore the use of adjusted SGA is more appropriate than an unadjusted centile or other methods with less evidence behind them. Another limitation is the relatively small sample size. Work is underway to gather a considerably larger cohort which will be used to further examine the findings.

The major strength of FMBV is that it is the only standardised PD measurement index which has been validated in 2D using radioactive microspheres in an animal model [17, 18]. Work is currently underway to replicate this study in 3D using our published technique [19]. The underlying image processing pipeline to calculate 3D FMBV currently requires a user to outline a number of slices of the placenta before the computer automatically measures the placental volume and extracts the UPI and VOIs of interest. Full automation of this process is well underway. This will allow placental volume and vascularity to be calculated in real-time, providing a promising imaging biomarker for adverse pregnancy outcome.

Our vision is that this simple to use tool will then be freely available to both the research and clinical community. This will facilitate large, multi-centre studies into the utility and effectiveness of these imaging markers alone and in combination with other known markers such as PAPP-A. This brings the possibility of developing a real-time first trimester imaging tool for pre-eclampsia and SGA significantly closer.

## Supporting information

S1 Dataset Excel spreadsheet of raw data for complete study cohort. (CSV)
